# Cutaneous dyspigmentation with perifollicular sparing

**DOI:** 10.1016/j.jdcr.2024.07.027

**Published:** 2024-08-13

**Authors:** Li Jie Helena Yoo, Sally McGrath, Odharnaith O’Brien, Siona Ní Raghallaigh

**Affiliations:** a Department of Dermatology, Beaumont Hospital, Dublin, Ireland; b Department of Pathology, Beaumont Hospital, Dublin, Ireland

**Keywords:** perifollicular sparing, salt and pepper dyspigmentation, systemic sclerosis

## History

**Figure 1:**
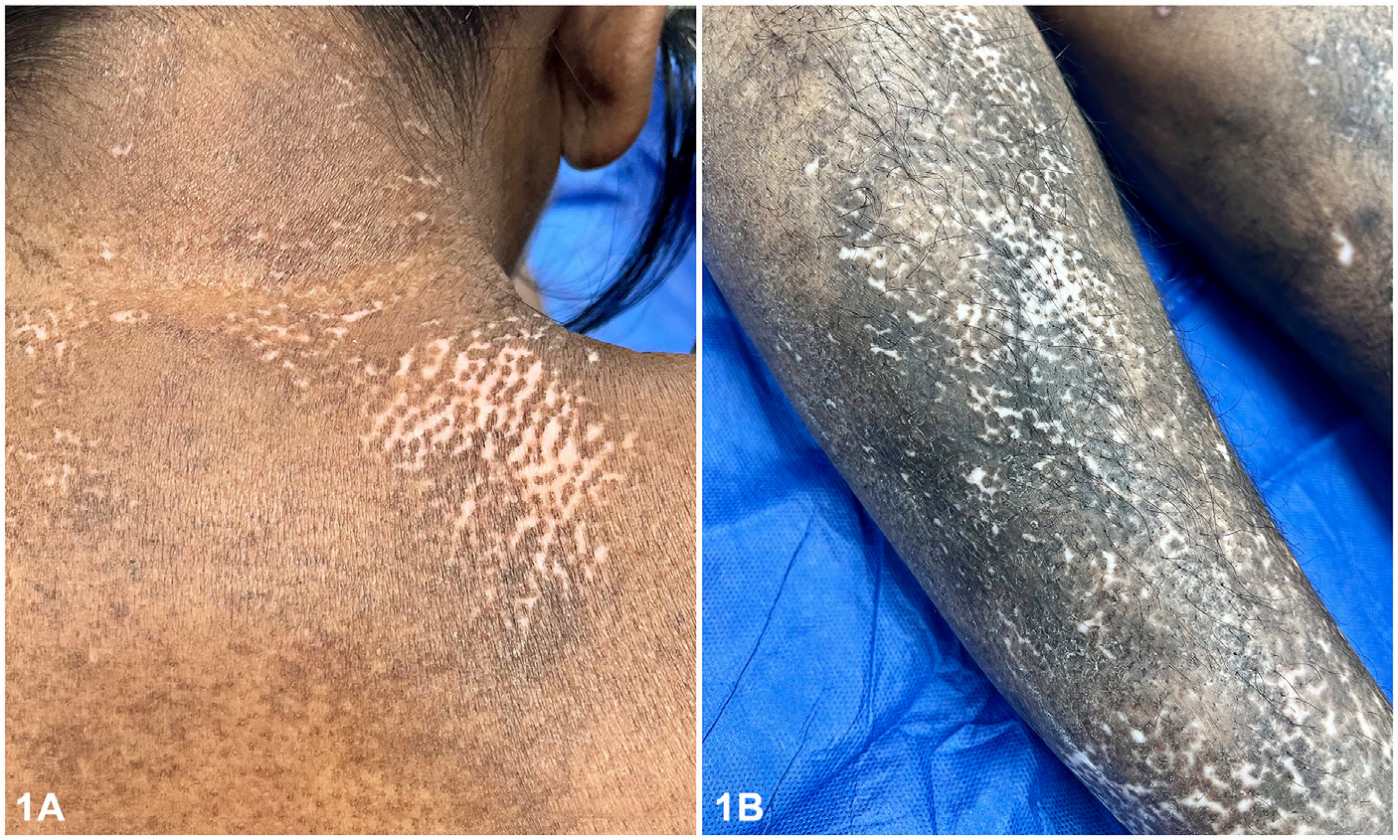

**Figure 2:**
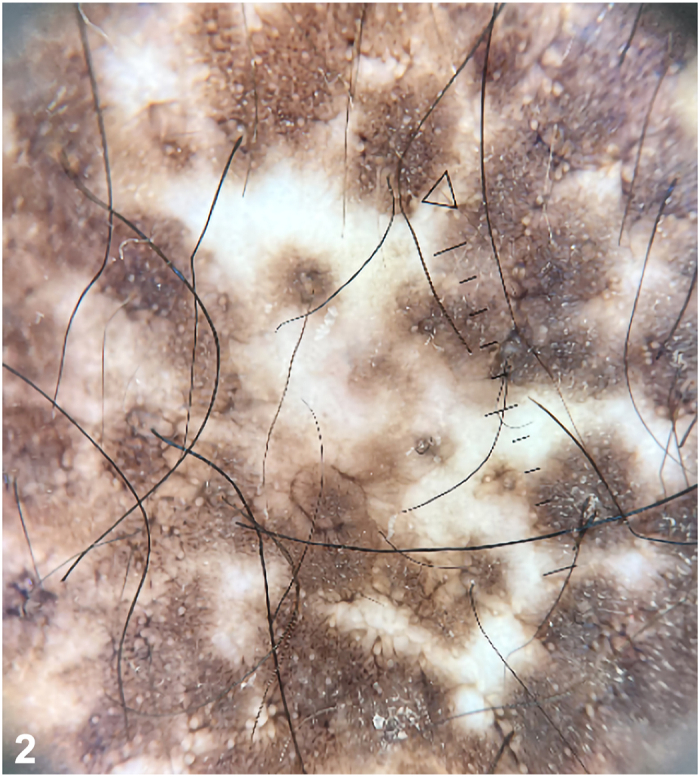

**Figure 3:**
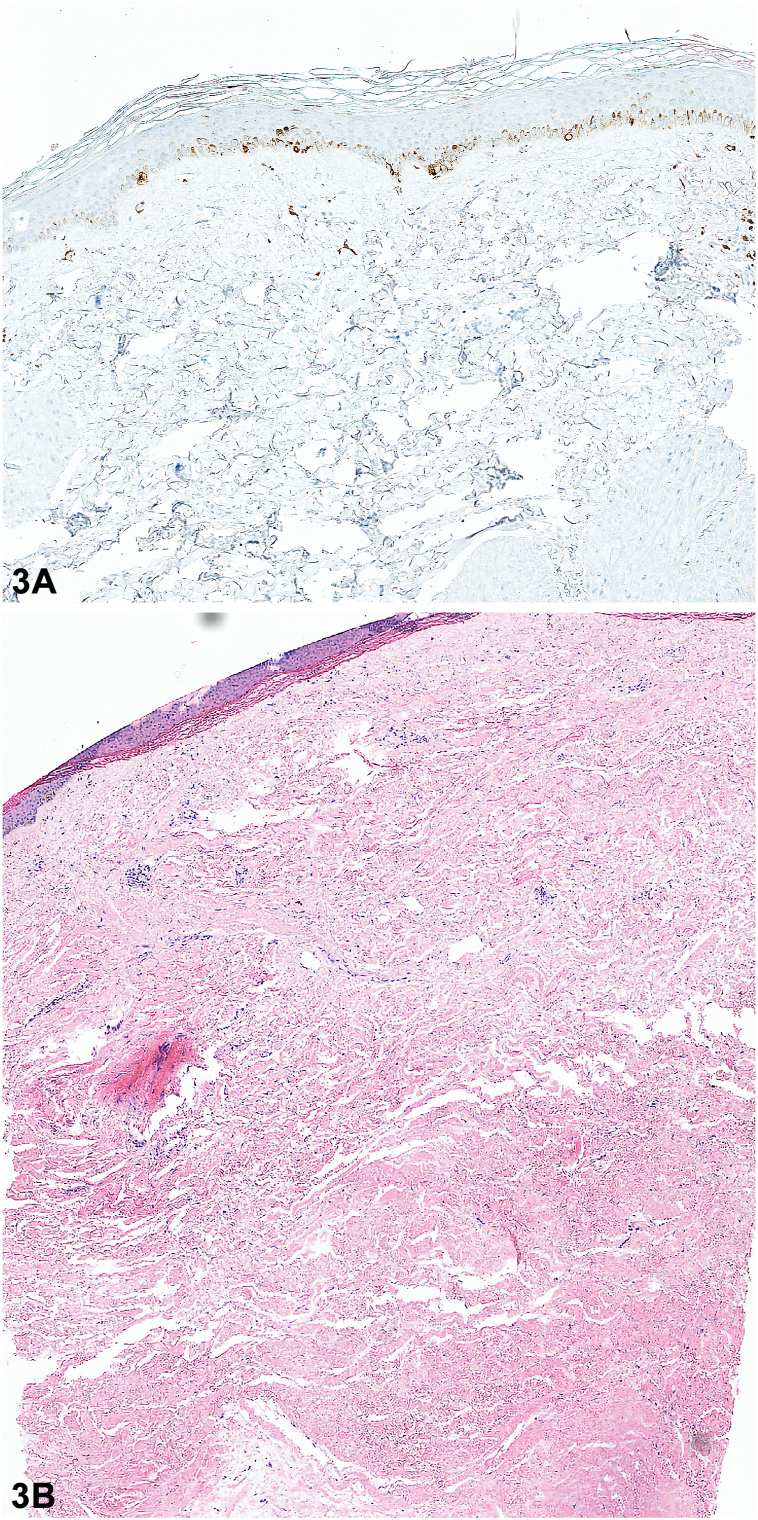

A 46-year-old woman presented to the emergency department with a 1-year history of polyarthralgia, dysphagia, unintentional weight loss, skin tightening, and depigmentation. Examination of the skin revealed areas of depigmentation on the back of the neck (Fig 1, *A*), bilateral upper portion of the arms, pretibial (Fig 1, *B*), associated with sclerodactyly, microstomia, and facial telangiectasia. Dermatoscopic examination of right pretibial depigmentation is shown in Fig 2. A punch biopsy of one of the depigmented area was performed and histologic findings are shown in Fig 3, *A, B*.

**Question 1: Which of the following is the most likely diagnosis?**

A. Vitiligo
B. Salt and pepper dyspigmentation associated with systemic sclerosis (SSc)
C. Hypertrophic lichen planus
D. Lichen sclerosus
E. Idiopathic guttate hypomelanosis

**Answers:**

A. Vitiligo – Incorrect. Histologic changes in vitiligo include absence of melanin in the dermoepidermal junction, no dermal pigments and a dermal lymphocytic infiltrate at the borders of depigmentation.^1^ On the contrary, histology findings in salt and pepper pattern from SSc demonstrate scattered clear cells containing melanin alternating with cells without melanin in the basal layer, melanophages in the papillary dermis (Fig 3, *A*) and dermal sclerosis (Fig 3, *B*).^1^
B. SSc – Correct. Skin hyperpigmentation and depigmentation (“salt and pepper”) is described in skin phenotypes IV to VI with SSc. Depigmentation in SSc are usually found in areas with skin hardening and diffuse hyperpigmentation (Fig 1, *B*).^1^ This patient had a remote history of digital ulceration, which resulted in digit shortening. Gastroscopy demonstrated chronic reflux esophagitis resulting in esophageal stricture formation, causing weight loss. Her antinuclear antibody showed a centromere pattern and histopathology was supportive.
C. Hypertrophic lichen planus – Incorrect. This is characterized by pruritic papules or plaques typically affecting the lower portion of the legs.
D. Lichen sclerosus – Incorrect. This superficial fungal infection caused by *Malassezia* typically presents with hypo- or hyper-pigmented macules on the trunk, which coalesce into large patches with fine scales. This condition does not typically affect the lower extremities.
E. Idiopathic guttate hypomelanosis – Incorrect. This common hypopigmentation disorder affects middle to older aged individuals with fair skin types. It is characterized by multiple, oval shaped hypopigmented macules on sun-exposed extremities.

**Question 2: Which of the following dermatoscopic feature** **supports** **the diagnosis?**

A. Areas of depigmentation sparing perifollicular areas
B. Well-defined depigmentation with pseudopod extension
C. Structureless linear white strands
D. Perifollicular depigmentation
E. Faint pigment network with fine scales

**Answers:**

A. Areas of depigmentation sparing perifollicular areas – Correct. Sparing of the perifollicular areas is a distinctive feature found in all SSc patients with salt and pepper pigmentation (Fig 2).^2^ This may be because of the richer capillary network on perifollicular skin preserving melanogenesis therefore retaining pigments.^2^
B. Well-defined depigmentation with pseudopod extension – Incorrect. This is a feature of idiopathic guttate hypomelanosis. The dermatoscopic appearance of margins that are diagnostic of idiopathic guttate hypomelanosis are amoeboid (pseudopod-like extensions), petaloid (leaf-like extensions), feathery (feather-like striations), and nebuloid (indistinct margins).^3^
C. Structureless linear white strands – Incorrect. This and follicular keratinous plugs are dermatoscopic features found in late sclerotic stages of lichen sclerosus. The histology finding that correlates with this feature are the upper dermal fibrotic bands.^3^
D. Perifollicular depigmentation – Incorrect. This occurs in active vitiligo with preservation of interfollicular pigmentation. Other dermatoscopic markers of progressive vitiligo are multiple hypopigmented dots surrounding a primary lesion, micro-Koebnerization and depigmentation in a starburst pattern.^3^
E. Faint pigment network with fine scales – Incorrect. This is a characteristic feature of pityriasis versicolor. The double-edged scales in skin creases are accentuated when the skin is stretched.^3^

**Question 3: Which of the following is the most appropriate first-line treatment for depigmentation in SSc?**

A. Mycophenolate mofetil
B. Nifedipine
C. Tofacitinib
D. Methotrexate
E. UV phototherapy

**Answers:**

A. Mycophenolate mofetil – Correct. Mycophenolate mofetil is often used as first-line treatment for cutaneous manifestations in SSc. A case series demonstrated its efficacy with >75% improvement of salt and pepper depigmentation after 12 months of treatment with doses ranging 1.5 to 3 g/d.^4^
B. Nifedipine – Incorrect. This calcium channel blocker is used as a first-line treatment for Raynaud phenomenon in SSc. There are no studies in the current literature to support the use of nifedipine in the treatment of depigmentation associated with SSc.
C. Tofacitinib – Incorrect. The Janus kinase/signal transducers and activators of transcription pathway has been implicated as the pathogenesis of depigmentation in SSc, similar to vitiligo.^5^ The use of a Janus kinase inhibitor has shown to be successful in disease control and repigmentation in 1 case report of a patient with diffuse SSc.^5^ Given the lack of evidence, it would not be the most appropriate first-line treatment.
D. Methotrexate – Incorrect. There are no studies in the current literature to support the use of methotrexate in the treatment of salt and pepper depigmentation in SSc.
E. UV phototherapy – Incorrect. One case report hypothesized that their patient experienced “autorepigmentation” after exposure to natural sunlight.^6^ To the best of our knowledge, no studies have addressed the use of phototherapy in treating depigmentation in SSc.

## Conflicts of interest

None disclosed.
